# Workplace Violence Against Chinese Frontline Clinicians During the COVID-19 Pandemic and Its Associations With Demographic and Clinical Characteristics and Quality of Life: A Structural Equation Modeling Investigation

**DOI:** 10.3389/fpsyt.2021.649989

**Published:** 2021-04-15

**Authors:** Yuan Yang, Yue Li, Ying An, Yan-Jie Zhao, Ling Zhang, Teris Cheung, Brian J. Hall, Gabor S. Ungvari, Feng-Rong An, Yu-Tao Xiang

**Affiliations:** ^1^Unit of Psychiatry, Department of Public Health and Medicinal Administration, Faculty of Health Sciences, Institute of Translational Medicine, University of Macau, Macao, China; ^2^Center for Cognition and Brain Sciences, University of Macau, Macao, China; ^3^Institute of Advanced Studies in Humanities and Social Sciences, University of Macau, Macao, China; ^4^Department of Nursing, Beijing Tongren Hospital, Capital Medical University, Beijing, China; ^5^Department of Emergency Medicine, Beijing Tongren Hospital, Capital Medical University, Beijing, China; ^6^Beijing Key Laboratory of Mental Disorders, The National Clinical Research Center for Mental Disorders, The Advanced Innovation Center for Human Brain Protection, School of Mental Health, Beijing Anding Hospital, Capital Medical University, Beijing, China; ^7^School of Nursing, Hong Kong Polytechnic University, Hong Kong, China; ^8^New York University Shanghai, Shanghai, China; ^9^University of Notre Dame Australia, Fremantle, WA, Australia; ^10^Division of Psychiatry, School of Medicine, Graylands Hospital, University of Western Australia, Perth, WA, Australia

**Keywords:** clinician, COVID-19, frontline, workplace, violence

## Abstract

**Background:** Workplace violence is a major concern for clinicians worldwide. There has been little data on the epidemiology of workplace violence against frontline clinicians during the COVID-19 pandemic. This study examined the pattern of workplace violence and its association with quality of life (QOL) against frontline clinicians during the outbreak of COVID-19 pandemic in China.

**Methods:** A cross-sectional online study was conducted in China between March 15 and March 20, 2020. Frontline clinicians' experience with workplace violence was measured with six standardized questions derived from the Workplace Violence Scale, while anxiety, depressive, and insomnia symptoms, and QOL were measured using the General Anxiety Disorder Questionnaire, the Patient Health Questionnaire, the Insomnia Severity Index, and the World Health Organization Quality of Life Questionnaire, respectively. Univariate analyses, multivariable logistic regression analyses, and structural equation modeling (SEM) were conducted.

**Results:** A total of 15,531 clinicians completed the assessment; 2,878 (18.5, 95% CI = 17.92–19.14%) reported workplace violence during the outbreak of the COVID-19 pandemic (verbal violence: 16.1%; physical violence: 6.9%). According to multivariable models, key correlates of workplace violence were male gender, longer work experience, higher education level, smoking, working in the psychiatry or emergency department, working in tertiary hospitals, being involved in direct care of infected patients, having infected family/ friends/ colleagues, and frequently using social communication programs. Clinicians working in inpatient departments were less likely to report workplace violence compared to those working in outpatient departments. SEM analysis revealed that both violence and emotional disturbances (anxiety, depression, and insomnia) directly affected QOL (standardized direct effect = −0.031, and −0.566, respectively, *P* < 0.05), while emotional disturbances partly mediated the association between work violence and QOL (standardized indirect effect = −0.184, *P* < 0.05).

**Conclusion:** Frontline clinicians were vulnerable to workplace violence during the COVID-19 pandemic. Due to the negative impact of workplace violence on quality of care and clinicians' QOL, health authorities and policymakers should take effective measures to reduce workplace violence against clinicians.

## Introduction

In late January, 2020, the World Health Organization (WHO) declared the novel coronavirus disease (COVID-19) as an international public health emergency (1). In order to avoid rapid transmission of the disease and provide timely clinical services for confirmed and suspected cases, frontline clinicians played a critical role in early identification of infected patients, which often made them face overwhelming workload, long working hours and great psychological stress. Frontline clinicians were often exposed to an elevated risk of infection, fatigue, anxiety, depression, insomnia, emotional exhaustion, burnout, and even workplace violence (2–4). The COVID-19 pandemic increased the likelihood of domestic violence, harassment, and stigmatization against clinicians (5). A growing number of attacks against clinicians has been reported globally (6). According to the International Committee of the Red Cross, 611 incidents of violence took place against health facilities, ambulances, and staff between February and July 2020 (5). Attacks from patients and/or families were common as clinicians need to implement essential COVID-19 prevention and control measures, such as, quarantining confirmed/suspected patients, and banning family visits, both of which disrupt communications between staff and patients/families thereby increasing the risk of conflicts (6). Yet, little is known about the patterns and consequences of workplace violence against clinicians during the COVID-19 pandemic in China.

Workplace violence refers to any act/threat of physical violence, harassment, intimidation, or other threatening disruptive behavior that happens in a workplace (7). It includes verbal and physical violence from patients, relatives, and even co-workers in clinical settings (8, 9). In the past decade workplace violence has been gaining growing attention worldwide including in clinical settings. Clinical workplace violence is associated with adverse consequences, such as job dissatisfaction, decreased quality of patient care, medical errors, and mental health problems (10–12). Therefore, in order to develop preventive measures to offset the negative outcomes of workplace violence, it is important to understand its patterns and associated factors.

The prevalence of workplace violence against clinicians is a significant concern. For instance, 12.1% of US clinicians in emergency departments (ED) experienced at least one type of violence in the past year (13), while the corresponding figures were 44.6% in Hong Kong nurses (10), and 89.9% among ED clinicians in Beijing, China (14). A recent meta-analysis found that the lifetime prevalence of workplace violence was 79.8% in ED clinicians in China (8). To the best of our knowledge, little is known about the patterns of workplace violence in frontline clinicians during the COVID-19 pandemic. The impact of workplace violence on QOL among clinicians during the COVID-19 pandemic remains unclear.

This study examined the pattern of workplace violence against frontline clinicians during the COVID-19 pandemic with special reference to clinicians QOL. According to the Distress/Protection model of QOL (15), QOL is determined by the interaction between protective (e.g., good social, and family support) and distressing factors (e.g., emotional disturbances and physical discomfort). This model postulated that an individual's satisfaction with QOL decreases if the distress factors outweigh protective factors, and vice versa (15). It has been consistently found that clinicians with emotional disturbances report lower QOL than those without (3, 16, 17). Clinicians exposed to violence are likely to have lower QOL in both physical and mental domains (18). Exposure to violence exposure increases the risk of emotional disturbances, such as, burnout and depression (19, 20). It is reasonable to assume that experience of violence and emotional disturbances are potential distress factors that independently affect QOL, and violence might also influence QOL through emotional disturbances. Therefore, the hypotheses of this study were: (1) both workplace violence and emotional disturbances (anxiety, depressive, and insomnia symptoms) would be significantly associated with lower QOL in frontline clinicians; (2) emotional disturbances would mediate the association between workplace violence and QOL during the COVID-19 pandemic.

## Methods

### Study Setting and Sample

A cross-sectional online survey was jointly organized by the Psychiatry, Emergency Medicine, Ophthalmology, and Otolaryngology Sections of the Chinese Nursing Association between March 15 and March 20, 2020 in China. To avoid the transmission of COVID-19, an online survey was adopted. All data were collected by the Wenjuanxing program, which is a survey application embedded within Wechat, a frequently used social communication program in China with more than 1 billion users. Snowball convenience sampling was used. To be eligible, participants needed to fulfill the following criteria: (1) aged 18 years and above; (2) frontline clinicians including doctors, nurses, and nursing assistants working in clinical settings of the abovementioned four specialties during the COVID-19 pandemic in China; (3) ability to read and speak Chinese; and (4) willingness to provide written informed consent. The study protocol was approved by the Ethics Committee of Beijing Anding Hospital, China.

### Assessment Instruments

Data collection form was utilized to collect basic demographic information. Additionally, participants were asked to answer: (1) whether they have personal experience with the 2003 Severe Acute Respiratory Syndrome (SARS) outbreak; (2) whether they were directly engaged in clinical services for patients with COVID-19; (3) whether their family, friends, or colleagues were infected with COVID-19; and (4) whether there were 500 or more COVID-19 cases in the province they lived in, and (5) whether they frequently used social communication programs to retrieve COVID-19 relevant news/information.

Workplace violence since the COVID-19 outbreak (January 20, 2020) was evaluated by six standardized questions derived from the Chinese version of the Workplace Violence Scale (21): two items measured participants' experience of verbal workplace violence (item 1) and threats (item 2), while the remaining four items measured experience of physical violence (item 3: physical assault with no physical injury; item 4: physical pain, bruises, or scratches; item 5: open wounds, fractures, internal organs, or head injuries; and item 6: dysfunction or permanent disability). Each item has four response options regarding frequency ranging from 0 (“none”) to 3 (“three times and above”) (21).

Depressive symptoms were measured with the 9-item Patient Health Questionnaire (PHQ-9). The PHQ-9 is a commonly used self-report scale with the total score ranging from 0 to 27 (22). The Chinese version of the PHQ-9 demonstrated good psychometric properties, with internal consistency of 0.89 (23). The 7-item General Anxiety Disorder Questionnaire (GAD) was used to assess anxiety symptoms; its total score ranged from 0 to 21 (24). The GAD-7 has been translated and validated in China, with an Cronbach's alpha of 0.91 (25). The 7-item Insomnia Severity Index (ISI) was utilized to evaluate insomnia symptoms, with the total score from 0 to 28 (26). The Chinese version of ISI showed satisfactory psychometric properties (27). The 26-item World Health Organization Quality of Life Questionnaire (WHOQOL-BREF) was used to measure QOL covering physical health, psychological health, social relationship, and environment health domains (28). The global QOL was calculated by adding up the sum of the first two items of the WHOQOL-BREF. A higher score indicates higher QOL (29). The Chinese version WHOQOL-BREF has good psychometric properties (30).

### Statistical Analysis

Data analyses were performed using SPSS, Version 21.0 and AMOS 21.0 (SPSS Inc., Chicago, IL, USA). The Kolmogorov-Smirnov test evaluated normality of the data. Comparison of the sociodemographic and clinical variables between the “workplace violence” and “no workplace violence” groups was performed using two independent samples *t*-tests, Mann-Whitney *U*-tests, or chi-square tests, as appropriate. To examine the independent sociodemographic correlates of workplace violence, multivariable logistic regression analyses with the “Enter” method (i.e., entering all independent variables in the model simultaneously) was conducted. Workplace violence was the dependent variable, while variables with significant group differences in the univariate analyses were entered as independent variables. Analysis of covariance (ANCOVA) was used to compare anxiety, depression, insomnia, and QOL separately between the two groups after controlling for covariates (variables that differed significantly in univariate analyses). Level of significance was set at *P* < 0.05 (two-tailed).

**Figure 1:**
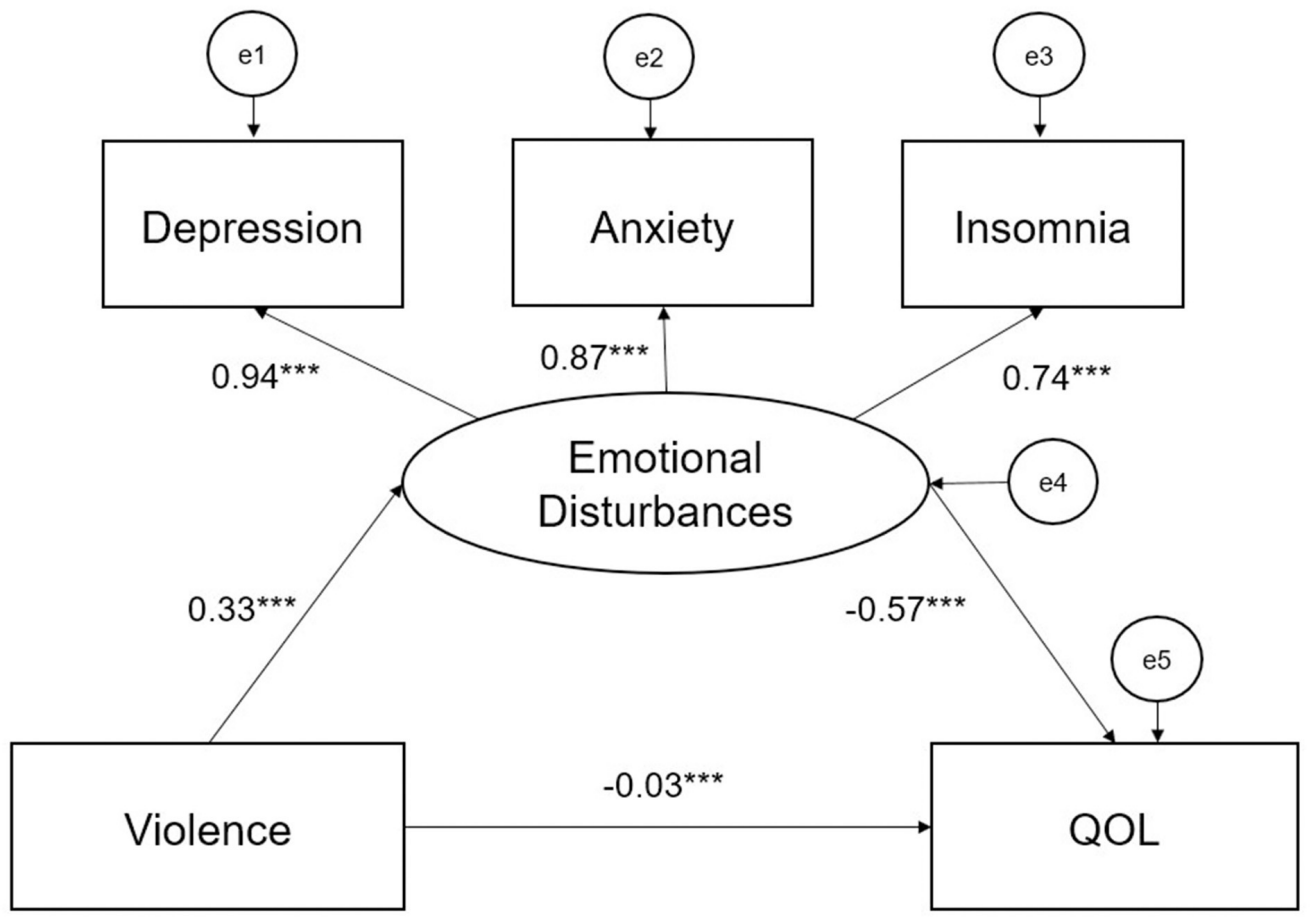
Violence on QOL by emotional disturbances. ***P < 0.001.

The direct and indirect associations between workplace violence, and psychological variables (anxiety, depression, and insomnia), and QOL were further examined with structural equation modeling (SEM) employing maximum likelihood estimation. Spearman's correlation analyses (rho) were conducted to examine bivariate associations among tested variables. PHQ, GAD, ISI, and QOL was entered as continuous variables in the model, and a latent (unobserved) variable of “emotional disturbances” was constructed to reflect the level of participants' anxiety, depressive, and insomnia symptoms (31). Instead of a complex model, a neat model was constructed because it is easier to interpret for clinicians with limited knowledge in statistics. Three different pathways were tested: (1) the path from workplace violence to QOL; (2) the path form emotional disturbances to QOL, and (3) the path from workplace violence to QOL mediated by emotional disturbances (Figure 1). The χ^2^/df, comparative fit index (CFI), normed-fit index (NFI), incremental fit index (IFI), Tucker-Lewis index (TLI), and Root Mean Square Error of Approximation (RMSEA) were considered as model fit indices (32). A higher than 0.90 of CFI, NFI, IFI, and TLI and a lower than 0.08 of RMSEA were indicative of good model fit (33).

## Results

A total of 15,531 participants completed the survey. The demographic and clinical characteristics of the sample are presented in Table 1. The mean age of the sample was 33.42 years (SD = 8.30); 1,770 (11.4%) were males. Altogether 2,878 (18.5, 95% CI = 17.92–19.14%) clinicians reported workplace violence during the COVID-19 pandemic: 2,507 (16.1%) were subjected to verbal and 1,066 (6.9%) to physical violence.

**Table 1:** Demographic characteristics of the study sample (N = 15,531).

**Variables**	**Total (*****N*** **=** **15,531)**		**No Violence (*****N*** **=** **12,653)**		**Violence (*****N*** **=** **2,878)**		**X^**2**^**	**df**	***P***
	***N***	**%**	***N***	**%**	***N***	**%**			
Gender (males)	1,770	11.4	1,343	10.6	427	14.8	41.402	1	** <0.001**
Married	10,912	70.3	8,887	70.2	2,025	70.4	0.018	1	0.895
College education and above	14,527	93.5	11,777	93.1	2,750	95.6	23.767	1	** <0.001**
Living with family	12,801	82.4	10,465	82.7	2,336	81.2	3.839	1	0.050
Department									
Psychiatry	10,516	67.7	8,568	67.7	1,948	67.7	106.462	3	**<0.001**
Emergency	1,103	7.1	781	6.2	322	11.2			
Ophthalmology	2,155	13.9	1,814	14.3	341	11.8			
Otolaryngology	1,757	11.3	1,490	11.8	267	9.3			
Experienced SARS in 2003	1,560	10.0	1,229	9.7	331	11.5	8.295	1	**0.004**
Working in tertiary hospital	10,803	69.6	8,708	68.8	2,095	72.8	17.469	1	**<0.001**
Working in inpatient department	13,159	84.7	10,878	86.0	2,281	79.3	81.710	1	**<0.001**
Shift duty	11,116	71.6	9,077	71.7	2,039	70.8	0.913	1	0.339
Number of local cases of COVID-19 patients >500	2,127	13.7	1,700	13.4	427	14.8	3.894	1	**0.048**
Having infected family/friends/colleagues	511	3.3	349	2.8	162	5.6	60.724	1	**<0.001**
Looking after infected patients	784	5.0	530	4.2	254	8.8	105.174	1	**<0.001**
Frequent communication program use	12,598	81.1	10,193	80.6	2,405	83.6	13.840	1	**<0.001**
Current smoker	272	1.8	169	1.3	103	3.6	68.570	1	**<0.001**
	**Mean**	**SD**	**Mean**	**SD**	**Mean**	**SD**	**T/Z**	**df**	***P***
Age (years)	33.42	8.30	33.33	8.34	33.80	8.08	−2.738	15529	**0.006**
Work experience (years)	11.89	9.03	11.80	9.07	12.33	8.86	−4.178	-^a^	**<0.001**
PHQ total score	3.48	4.41	2.86	3.88	6.23	5.45	−36.170	-^a^	**<0.001**
GAD total score	2.46	3.67	1.94	3.18	4.74	4.66	−36.754	-^a^	**<0.001**
ISI total score	4.67	4.89	4.07	4.50	7.34	5.61	−32.047	-^a^	**<0.001**
QOL total score	6.62	1.59	6.78	1.56	5.90	1.52	27.542	15529	**<0.001**

*Bolded values: < 0.05; M, mean; SD, standard deviation; COVID-19, Corona Virus Disease 2019; SARS, Severe acute respiratory syndrome; PHQ, Patient Health Questionnaire; GAD, Generalized Anxiety Disorder scale; ISI, Insomnia Severity Index; QOL, Quality of Life; a = Mann-Whitney U-test*.

Univariate analyses found that workplace violence was significantly associated with male sex (*P* < 0.001), older age (*P* = 0.006), longer work experience (*P* < 0.001), higher education level (*P* < 0.001), department (i.e., specialty) (*P* < 0.001), experience with the 2003 SARS epidemic (*P* = 0.004), working in tertiary hospitals (*P* < 0.001), working in inpatient departments (*P* < 0.001), looking after infected patients (*P* < 0.001), having infected family/friends/colleagues (*P* < 0.001), more than 500 confirmed cases in the province (*P* = 0.048), frequent use of communication programs (*P* < 0.001), and smoking (*P* < 0.001). Participants having experienced workplace violence also reported more anxiety, depression and insomnia symptoms, and lower QOL score (all *P* < 0.001) (Table 1).

Multivariable logistic regression analysis revealed that workplace violence was positively associated with male sex (OR = 1.382, *P* < 0.001), longer work experience (OR = 1.006, *P* = 0.009), higher education level (OR = 1.691, *P* < 0.001), working in psychiatric or ED (OR = 1.398, and 1.655, respectively, *P* < 0.001), working in tertiary hospitals (OR = 1.178, *P* = 0.001), looking after infected patients (OR = 1.834, *P* < 0.001), having family/friends/colleagues infected with COVID-19 (OR = 1.733, *P* < 0.001), frequent use of communication programs (OR = 1.210, *P* = 0.001) and smoking (OR = 2.366, *P* < 0.001). Clinicians working in inpatient departments were less likely to report workplace violence compared to those working in outpatient departments (OR = 0.680, *P* < 0.001). After controlling for covariates, ANCOVA showed that workplace violence was significantly associated with more severe anxiety, depression, and insomnia symptoms, and lower QOL score (all *P* < 0.001) (Table 2).

**Table 2:** Correlates of violence by multiple logistic regression analysis and ANCOVA.

**Variables**	**Multiple logistic regression analysis**		
	**OR**	**95% CI**	***P*-value**
Gender (males)	1.382	1.215–1.570	**<0.001**
College education and above	1.691	1.391–2.056	**<0.001**
Department			
Psychiatry	1.398	1.209–1.616	**<0.001**
Emergency	1.655	1.354–2.022	**<0.001**
Ophthalmology	1.012	0.848–1.208	0.892
Otolaryngology	Ref	-	-
Experienced SARS in 2003	1.061	0.927–1.214	0.394
Working in tertiary hospital	1.178	1.072–1.295	**0.001**
Working in inpatient department	0.680	0.603–0.767	**<0.001**
Number of local cases of COVID-19 patients >500	1.038	0.923–1.168	0.534
Having infected family/friends/colleagues	1.733	1.417–2.119	**<0.001**
Looking after infected patients	1.834	1.556–2.183	**<0.001**
Frequent communication program use	1.210	1.084–1.351	**0.001**
Current smoker	2.366	1.822–3.073	**<0.001**
Work experience (years)	1.006	1.002–1.011	**0.009**
	**ANCOVA**		
	**F**	**df**	***P***
PHQ total score	1397.668	1	**<0.001**^a^
GAD total score	1411.725	1	**<0.001**^a^
ISI total score	1023.693	1	**<0.001**^a^
Overall QOL score	707.918	1	**<0.001**^a^

*Bold values: P < 0.05; CI, confidential interval; OR, odds ratio; COVID-19, Corona Virus Disease 2019; SARS, Severe acute respiratory syndrome; PHQ, Patient Health Questionnaire; GAD, Generalized Anxiety Disorder scale; ISI, Insomnia Severity Index; QOL, Quality of Life; a, using gender, education, department, 2003 SARS experience, working in tertiary hospital, working in inpatient department, number of COVID-19 patients >500, having infected family/friends/colleagues, nursing care for infected patients, frequent communication program use, currently smoking and working experience as covariates*.

### Structural Equation Modeling (SEM)

The results of Spearman correlation analyses are shown in Table 3. Workplace violence was positively associated with more severe anxiety (*r* = 0.295, *P* < 0.001), depressive (*r* = 0.290, *P* < 0.001), and insomnia symptoms (*r* = 0.257, *P* < 0.001), and negatively associated with QOL scores (*r* = −0.220, *P* < 0.001). Anxiety, depressive, and insomnia symptoms were also significantly associated with lower QOL (r = −0.518, −0.559, and −0.534; all *P* < 0.001). Figure 1 presents the model of the association between workplace violence and QOL mediated by emotional disturbances (anxiety, depressive, and insomnia symptoms). SEM analysis found that the model had a decent fit (χ^2^/df = 7.883, CFI = 0.975, NFI = 0.975, TLI = 0.960, IFI = 0.975, and RMSEA = 0.067) after controlling for age and sex. Frontline clinicians' experience of workplace violence directly affected QOL, and emotional disturbances partly mediated the association between workplace violence and QOL. The standardized total effect of workplace violence on QOL was −0.215 (standardized direct effect = −0.031, *P* < 0.05; standardized indirect effect = −0.184, *P* < 0.05). Emotional disturbances also directly affected QOL. The standardized total effect of emotional disturbances on QOL was −0.566 (*P* < 0.05).

**Table 3:** Key variables and Spearman correlation coefficients.

	**1**	**2**	**3**	**4**	**5**
1. Workplace violence	1				
2. PHQ Total Score	0.290^***^	1			
3. GAD Total Score	0.295^***^	0.774^***^	1		
4. ISI Total Score	0.257^***^	0.682^***^	0.597^***^	1	
5. QOL total score	−0.220^***^	−0.559^***^	−0.518^***^	−0.534^***^	1

*** *P < 0.001; PHQ, Patient Health Questionnaire; GAD, Generalized Anxiety Disorder scale; ISI, Insomnia Severity Index; QOL, Quality of Life*.

## Discussion

In this study, 18.5% (95% CI = 17.92–19.14%) of frontline clinicians reported workplace violence about 2 months after the outbreak of the COVID-19 pandemic. Since this is the first study that estimated the prevalence of workplace violence during COVID-19, no comparison to other similar investigations is possible. In a meta-analysis the pooled 12-month prevalence of workplace violence against clinicians was 58.7% (95% CI = 46.0–71.4%) in North America, 45.5% (95% CI = 40.4–50.7%) in Asia, and 31.6% (95% CI = 27.1–36.1%) in selected European countries. Another meta-analysis found that the lifetime prevalence of workplace violence was 79.8% in ED clinicians in China (8). However, caution is warranted as the study samples and timeframes are not directly comparable.

The causes of workplace violence against clinicians are complex. Many clinicians volunteered to work in designated hospitals, which increased pressure on already limited health resources. Consequently, patients and families were often dissatisfied with limited access to medical care, crowded treatment environment, long waiting hours, and insufficient communication with clinicians, all of which raised the likelihood of workplace violence against frontline clinicians (34). In addition, frontline clinicians faced great pressure and overwhelming workload during the COVID-19 pandemic exacerbating their exhaustion and emotional disturbances, and affecting communication with patients and families (35). Furthermore, the public's fears, worries, and discrimination against those who were likely to increase disease transmission may have also escalated the risks of violence against frontline clinicians (36–38).

Consistent with previous studies (39, 40), male frontline clinicians were more likely to experience workplace violence than their female counterparts in this survey. Male clinicians are more likely to experience physical violence, but less likely to be subjected to sexual harassment than female clinicians (35). Contrary to the findings of the current study, no significant association between education and workplace violence was found in previous studies (35). Investigations about the association between smoking and workplace violence yielded conflicting results. Nurses who smoked were more likely to experience workplace violence (41), but this finding was not confirmed (42). In this study, smoking clinicians were more likely to face workplace violence than their non-smoking colleagues. The reason behind the mixed findings across studies is probably explained by different sociocultural contexts, specialty, sample size, sampling method, and definitions on workplace violence, smoking, and violence.

Compared to in other specialties, the prevalence of workplace violence was higher in psychiatry and ED in this study, which is consistent with previous findings (39, 43). Many mental health and ED clinicians volunteered to work in infectious disease hospitals in crisis response teams, which increased pressure on existing scant health resources in their original hospitals. Low clinician-to-patient ratio, together with many patients' worsening symptoms in psychiatric settings, or life-threatening conditions in ED requiring immediate attention (34, 44), affected the efficiency and quality of care, and possibly increased patients' and their families' dissatisfaction and irritability, eventually leading to conflicts with clinicians, and subsequent violent acts (34, 35). In line with previous studies, clinicians with longer work experience were more likely to encounter workplace violence (35). It is possible that more experienced clinicians are exposed to more difficult and challenging patients and caregivers than junior staff, which increase the likelihood of a violent incident.

In this study, frontline clinicians who worked in tertiary hospitals, were more likely to experience workplace violence than those in secondary hospitals and community clinics in China, confirming previous findings (35, 45). Secondary hospitals or community settings have higher clinician-patient ratio and less severe cases, which reduces the likelihood of workplace violence. Clinicians working in inpatient setting were less likely to suffer workplace violence than those in outpatient setting. It is possible that clinicians in inpatient settings have more time to communicate with patients, and to provide more timely clinical services (46, 47).

Variables relevant to COVID-19, including caring for infected patients, having family, or friends infected with COVID-19, and frequent use of social communication programs to retrieve information on COVID-19, were significantly associated with higher likelihood of workplace violence. Due to the fears and concern of COVID-19 transmission in hospitals, many frontline clinicians were verbally, and even sometimes physically abused by the public as “disease spreaders” in the early stage of the COVID-19 pandemic (48) as they were wrongfully considered as the vectors of contagion in the community (36). In addition, the overwhelmingly negative or false news on COVID-19 exacerbated the public's fear of contagion and psychological stress (2, 3). Clinicians with high levels of psychological stress are more prone to medical errors and poorer interpersonal communication with patients and their families, which put them at higher risk for violence.

SEM analysis confirmed the study hypothesis that both workplace violence and emotional disturbances would directly affect QOL, while emotional disturbances significantly mediate the association between violence and QOL. Previous studies found workplace violence to be a significant contributor to clinicians' lower QOL (11, 18), and suggested that the implementation of violence prevention measures and policies would be beneficial to improve their QOL. There is a positive correlation between workplace violence and anxiety, depression, and insomnia symptoms (11, 49, 50). Individuals exposed to workplace violence are more likely to suffer from impaired psychological adjustment, poor work performance, and social interactions with others (51), which lead to emotional disturbances and even self-harm and suicide (51).

The strengths of this study are the large sample size and the use of sophisticated statistical analyses. However, several limitations need to be noted. First, snowball sampling and the unequal sex composition of the sample - most participants were females - constituted selection bias. Furthermore, psychiatric clinicians accounted for the majority of the participants. Second, several factors associated with workplace violence, such as, clinician-patient relationship, and participants' preexisting psychological or psychiatric conditions, were not investigated. Third, due to the cross-sectional design, the causal associations between variables and violence are still unknown. Fourth, only clinicians in four specialties were examined, therefore, the findings cannot be generalized to all frontline clinicians. Fifth, due to the online snowball convenience sampling, the response rate could not be calculated. Finally, workplace violence was measured by self-rated standardized questions. Further studies should rely on more objective measurement.

In conclusion, frontline clinicians were vulnerable to experience workplace violence during the early days of the COVID-19 pandemic. Due to the negative impact of workplace violence on the quality of care and frontline clinicians' QOL, health authorities and policymakers should devise effective measures to reduce workplace violence against clinicians.

## Data Availability Statement

The Clinical Research Ethics Committee of Beijing Anding Hospital that approved the study prohibits the authors from making the research data set publicly available. Readers and all interested researchers may contact Dr. Feng-Rong An (afrylm@sina.com) for details. Dr. An could apply to the Clinical Research Ethics Committee of Beijing Anding Hospital for the release of the data.

## Ethics Statement

The studies involving human participants were reviewed and approved by the Ethics Committee of Beijing Anding Hospital. The patients/participants provided their written informed consent to participate in this study.

## Author Contributions

YL, YA, F-RA, and Y-TX: study design. YY, YL, YA, Y-JZ, and LZ: data collection, analysis, and interpretation. YY, TC, and Y-TX: drafting of the manuscript. BH and GU: critical revision of the manuscript. All co-authors: approval of the final version for publication. All authors contributed to the article and approved the submitted version.

## Conflict of Interest

The authors declare that the research was conducted in the absence of any commercial or financial relationships that could be construed as a potential conflict of interest.

## References

[1] World Health Organization. The Coronavirus Disease (COVID-19) Outbreak. (2020). Available online at: https://wwwwhoint (accessed March 30, 2020).

[2] Hu D Kong Y Li W Han Q Zhang X Zhu LX. Frontline nurses' burnout, anxiety, depression, and fear statuses and their associated factors during the COVID-19 outbreak in Wuhan, China: a large-scale cross-sectional study. EClinicalMedicine. (2020) 24:100424. 10.1016/j.eclinm.2020.100424 32766539 PMC7320259

[3] Lai J Ma S Wang Y Cai Z Hu J Wei N. Factors associated with mental health outcomes among health care workers exposed to coronavirus disease 2019. JAMA Netw Open. (2020) 3:e203976. 10.1001/jamanetworkopen.2020.3976 32202646 PMC7090843

[4] Zhao K Zhang G Feng R Wang W Xu D Liu Y. Anxiety, depression and insomnia: a cross-sectional study of frontline staff fighting against COVID-19 in Wenzhou, China. Psychiatry Res. (2020) 292:113304. 10.1016/j.psychres.2020.113304 32731081 PMC7361101

[5] Devi S. COVID-19 exacerbates violence against health workers. Lancet. (2020) 396:658. 10.1016/S0140-6736(20)31858-4 32891198 PMC7470723

[6] Forgione P. New Patterns of Violence Against Healthcare in the Covid-19 Pandemic. (2020). Available online at: https://blogsbmjcom/bmj/2020/05/15/new-patterns-of-violence-against-healthcare-in-the-covid-19-pandemic/ (accessed Febraury 5, 2021).

[7] National Institute for Occupational Safety and Health. Understanding Workplace Violence Prevention and Response. (2020). Available online at: https://ssoshrmorg/IDBUS/SHRM/PORTAL-EE/JOSSO/SSO/REDIR?josso_cmd=login&josso_partnerapp_id=portal-sp (accessed August 31, 2020).

[8] Lu L Dong M Wang SB Zhang L Ng CH Ungvari GS. Prevalence of workplace violence against health-care professionals in china: a comprehensive meta-analysis of observational surveys. Trauma Violence Abuse. (2020) 21:498–509. 10.1177/1524838018774429 29806556

[9] Jiao M Ning N Li Y Gao L Cui Y Sun H. Workplace violence against nurses in Chinese hospitals: a cross-sectional survey. BMJ Open. (2015) 5:e006719. 10.1136/bmjopen-2014-006719 25814496 PMC4386227

[10] Cheung T Yip PS. Workplace violence towards nurses in Hong Kong: prevalence and correlates. BMC Public Health. (2017) 17:196. 10.1186/s12889-017-4112-3 28196499 PMC5310001

[11] Wu S Lin S Li H Chai W Zhang Q Wu Y. A study on workplace violence and its effect on quality of life among medical professionals in China. Arch Environ Occup Health. (2014) 69:81–8. 10.1080/19338244.2012.732124 24205959

[12] Hall BJ Xiong P Chang K Yin M Sui XR. Prevalence of medical workplace violence and the shortage of secondary and tertiary interventions among healthcare workers in China. J Epidemiol Commun Health. (2018) 72:516–8. 10.1136/jech-2016-208602 29475953

[13] Speroni KG Fitch T Dawson E Dugan L Atherton M. Incidence and cost of nurse workplace violence perpetrated by hospital patients or patient visitors. J Emerg Nurs. (2014) 40:218–28; quiz 95. 10.1016/j.jen.2013.05.014 24054728

[14] Li N Zhang L Xiao G Chen J Lu Q. The relationship between workplace violence, job satisfaction and turnover intention in emergency nurses. Int Emerg Nurs. (2019) 45:50–5. 10.1016/j.ienj.2019.02.001 30797732

[15] Voruganti L Heslegrave R Awad AG Seeman MV. Quality of life measurement in schizophrenia: reconciling the quest for subjectivity with the question of reliability. Psychol Med. (1998) 28:165–72. 10.1017/S0033291797005874 9483693

[16] Singleton SS. Depression and quality of life: a patient's perspective. J Clin Psychiatry. (2001) 62(Suppl. 26):22. 11775089

[17] Ivbijaro G Kolkiewicz L Goldberg D Riba MB N'Jie I NS Geller J. Preventing suicide, promoting resilience: is this achievable from a global perspective? Asia Pac Psychiatry. (2019) 11:e12371. 10.1111/appy.12371 31709743

[18] Zeng JY An FR Xiang YT Qi YK Ungvari GS Newhouse R. Frequency and risk factors of workplace violence on psychiatric nurses and its impact on their quality of life in China. Psychiatry Res. (2013) 210:510–4. 10.1016/j.psychres.2013.06.013 23850435

[19] Li P Xing K Qiao H Fang H Ma H Jiao M. Psychological violence against general practitioners and nurses in Chinese township hospitals: incidence and implications. Health Qual Life Outcomes. (2018) 16:117. 10.1186/s12955-018-0940-9 29871642 PMC5989437

[20] Yan F Tang SY Goldsamt L Wang HH Chen J Li XH. Interrelationships between intimate partner violence, coping style, depression, and quality of life among the regular female sexual partners of men who have sex with men. J Interpers Violence. (2020) 11:1–20. 10.1177/0886260520917519 32390497

[21] Chen ZH Wang SY Lu YC Jing CX. Analysis on the epidemiological features and risk factors of hospital workplace violence in Guangzhou (in Chinese). Zhonghua Liu Xing Bing Xue Za Zhi. (2004) 25:3–5. 15061937

[22] Kroenke K Spitzer RL Williams JB Lowe B. The patient health questionnaire somatic, anxiety, and depressive symptom scales: a systematic review. Gen Hosp Psychiatry. (2010) 32:345–59. 10.1016/j.genhosppsych.2010.03.006 20633738

[23] Chen M Sheng L Qu S. Diagnostic test of screening depressive disorder in general hospital with the Patient Health Questionnaire (in Chinese). Chin Ment Health. (2015) 29:241–5.

[24] Spitzer RL Kroenke K Williams JB Lowe B. A brief measure for assessing generalized anxiety disorder: the GAD-7. Arch Intern Med. (2006) 166:1092–7. 10.1001/archinte.166.10.1092 16717171

[25] Zheng Q. Reliability and validity of Chinese version of Generalized Anxiety Disorder 7-item (GAD-7) scale in screening anxiety disorder in outpatients from traditional Chinese internal department (in Chinese). Chin Ment Health. (2013) 27:163–8.

[26] Bastien CH Vallieres A Morin CM. Validation of the insomnia severity index as an outcome measure for insomnia research. Sleep Med. (2001) 2:297–307. 10.1016/S1389-9457(00)00065-4 11438246

[27] Yu DS. Insomnia severity index: psychometric properties with Chinese community-dwelling older people. J Adv Nurs. (2010) 66:2350–9. 10.1111/j.1365-2648.2010.05394.x 20722803

[28] Harper A Power M Grp W. Development of the World Health Organization WHOQOL-BREF quality of life assessment. Psychol Med. (1998) 28:551–8. 10.1017/S0033291798006667 9626712

[29] Skevington SM Tucker C. Designing response scales for cross-cultural use in health care: data from the development of the UK WHOQOL. Br J Med Psychol. (1999) 72:51–61. 10.1348/000711299159817 10194572

[30] Fang JQ Hao YA. Reliability and validity for chinese version of WHO quality of life scale (in Chinese). Chin Ment Health J. (1999) 13:203–9.

[31] Zhang WJ Yan C Shum D Deng CP. Responses to academic stress mediate the association between sleep difficulties and depressive/anxiety symptoms in Chinese adolescents. J Affect Disord. (2020) 263:89–98. 10.1016/j.jad.2019.11.157 31818801

[32] Bentler PM. Fit indexes, lagrange multipliers, constraint changes, and incomplete data in structural models. Multivariate Behav Res. (1990) 25:163–72. 10.1207/s15327906mbr2502_3 26794478

[33] Bentler PM. Comparative fit indexes in structural models. Psychol Bull. (1990) 107:238–46. 10.1037/0033-2909.107.2.238 2320703

[34] Chen S Lin S Ruan Q Li H Wu S. Workplace violence and its effect on burnout and turnover attempt among Chinese medical staff. Arch Environ Occup Health. (2016) 71:330–7. 10.1080/19338244.2015.1128874 26654585

[35] Liu J Gan Y Jiang H Li L Dwyer R Lu K. Prevalence of workplace violence against healthcare workers: a systematic review and meta-analysis. Occup Environ Med. (2019) 76:927–37. 10.1136/oemed-2019-105849 31611310

[36] World Health Organization. Attacks on Health Care in the Context of COVID-19. (2020). Available online at: https://wwwwhoint/news-room/feature-stories/detail/attacks-on-health-care-in-the-context-of-covid-19 (accessed September 1, 2020).

[37] Venugopal VC Mohan A Chennabasappa LK. Status of mental health and its associated factors among the general populace of India during COVID-19 pandemic. Asia Pac Psychiatry. (2020) 24:e12412. 10.1111/appy.12412 32830876 PMC7460994

[38] Ghosh A. COVID-19 pandemic and an early career mental health researcher from a low and middle income country: Is there any light at the end of the tunnel? Asia Pac Psychiatry. (2020) 12:e12424. 10.1111/appy.12424 32949215 PMC7536927

[39] Campbell JC Messing JT Kub J Agnew J Fitzgerald S Fowler B. Workplace violence: prevalence and risk factors in the safe at work study. J Occup Environ Med. (2011) 53:82–9. 10.1097/JOM.0b013e3182028d55 21187791

[40] Gan Y Li L Jiang H Lu K Yan S Cao S. Prevalence and risk factors associated with workplace violence against general practitioners in Hubei, China. Am J Public Health. (2018) 108:1223–6. 10.2105/AJPH.2018.304519 30024800 PMC6085021

[41] Arnetz JE Arnetz BB Petterson IL. Violence in the nursing profession: occupational and lifestyle risk factors in Swedish nurses. Work Stress. (1996) 10:119–27. 10.1080/02678379608256791

[42] Yang YJ Moon YH Do SY Lee CG Song HS. Effects of work-related factors on self-reported smoking among female workers in call centers: a cross-sectional study. Ann Occup Environ Med. (2019) 31:4. 10.1186/s40557-019-0286-8 30805195 PMC6373141

[43] Gerberich SG Church TR McGovern PM Hansen H Nachreiner NM Geisser MS. Risk factors for work-related assaults on nurses. Epidemiology. (2005) 16:704–9. 10.1097/01.ede.0000164556.14509.a3 16135952

[44] Ajani K. Triage: a literature review of key concepts. J Pak Med Assoc. (2012) 62:487–9. 22755315

[45] Jatic Z Erkocevic H Trifunovic N Tatarevic E Keco A Sporisevic L. Frequency and forms of workplace violence in primary health care. Med Arch. (2019) 73:6–10. 10.5455/medarh.2019.73.6-10 31097851 PMC6445619

[46] Fleischman RJ Kaji AH Diaz VM McKenzie K Soltero P Van Natta TL. A simple intervention to improve hospital flow from emergency department to inpatient units. JAMA Intern Med. (2015) 175:289–90. 10.1001/jamainternmed.2014.6689 25545454

[47] Tuna O Enez Darcin A Tarakcioglu MC Aksoy UM. COVID-19 positive psychiatry inpatient unit: a unique experience. Asia Pac Psychiatry. (2020) 12:e12410. 10.1111/appy.12410 32812352 PMC7460997

[48] Nursing Times. Nurses on Coronavirus Frontline Facing ‘Abhorrent' Abuse from Public. (2020). Available online at: https://wwwnursingtimesnet/news/coronavirus/nurses-fighting-coronavirus-facing-abhorrent-abuse-from-public-20-03-2020/ (accessed September 1, 2020).

[49] Duan X Ni X Shi L Zhang L Ye Y Mu H. The impact of workplace violence on job satisfaction, job burnout, and turnover intention: the mediating role of social support. Health Qual Life Outcomes. (2019) 17:93. 10.1186/s12955-019-1164-3 31146735 PMC6543560

[50] Gacki-Smith J Juarez AM Boyett L Homeyer C Robinson L MacLean SL. Violence against nurses working in US emergency departments. J Nurs Adm. (2009) 39:340–9. 10.1097/NNA.0b013e3181ae97db 19641432

[51] Bordignon M Monteiro MI. Violence in the workplace in nursing: consequences overview. Rev Bras Enferm. (2016) 69:99–9. 10.1590/0034-7167-2015-0133 27783746

